# Effect of Environment on Acetylated Cellulose Nanocrystal-Reinforced Biopolymers Films

**DOI:** 10.3390/polym15071663

**Published:** 2023-03-27

**Authors:** Ana Oberlintner, Blaž Likozar, Uroš Novak

**Affiliations:** 1 Department of Catalysis and Chemical Reaction Engineering, National Institute of Chemistry, SI-1000 Ljubljana, Slovenia; ana.oberlintner@ki.si; 2 International Postgraduate School Jožef Stefan, SI-1000 Ljubljana, Slovenia; blaz.likozar@ki.si

**Keywords:** functionalisation, acetylation, reinforcing cellulose nanocrystal, bionanocomposites, biopolymer-based composite films, bio-based flexible packaging

## Abstract

Cellulose nanocrystals (CNCs) were acetylated to the various parametrised degrees of substitution (DS), determined through attenuated total reflection Fourier transform infrared spectroscopy (ATR–FTIR) and incorporated into alginate (ALG) and chitosan (CH) film-forming solutions. An investigation of morphology with scanning electron microscopy (SEM) revealed increased chemical compatibility with the CH matrix after acetylation, producing a smooth surface layer, while ALG mixed better with pristine CNCs. The ATR–FTIR analysis of films demonstrated inter-diffusional structural changes upon the integration of pristine/modified CNCs. Films were evaluated in terms of water contact angle (WCA), which decreased upon CNC addition in either of the biocomposite types. The H_2_O barrier assessed through applicative vapour transmission (WVT) rate increased with the CNC esterification in CH, but was not influenced in ALG. To evaluate the relationship between environmental humidity and mechanical properties, conditioning was applied for 48 h under controlled relative humidity (33%, 54% and 75%) prior to the evaluation of the mechanical properties and moisture content. It was observed that tensile strength was highest upon specimens being dry (25 ± 3 MPa for ALG, reinforced with neat CNCs, or 16 ± 2 MPa in the CH with CNCs, reacting to the highest DS), lowering with dewing, and the elongation at break exhibited the opposite. It is worth noting that the modification of CNCs improved the best base benchmark stress–strain performance. Lastly, (thermal) stability was assessed by means of the thermogravimetric analysis (TGA) technique, suggesting a slight improvement.

## 1. Introduction

In light of recent efforts to find a suitable alternative that would be indispensable in the fight against plastic pollution, marine biomass has become an attractive raw-material source of biopolymers [1]. Considering that the packaging sector uses a noteworthy amount of all produced plastics (44% in the EU market in 2021 [2]), along with the fact that these materials also have the shortest lifetime, as it is limited to the lifetime of its contents (e.g., fresh produce), bio-based and biodegradable packaging materials are urgently needed to ensure a cleaner future. One of the solutions is offered by chitosan, a derivate of chitin—the second most abundant polymer on the planet. Chitin is found in the shells of crustaceans, a waste product of the food-processing industry, the use of which is in line with circular-economy guidelines. The disadvantage of marine waste biomass as a raw material is tropomyosin, a known allergen found in the muscles of crustaceans, leading to the popularization of fungal biomass as a source of chitin [3]. Chitosan is obtained from chitin using (partial) deacetylation, and therefore consists of β-D-1,4-glucosamine and N-acetyl-D-glucosamine units linked by glycosidic bonds. Chitosan is, contrary to chitin, soluble in acidic aqueous media and exhibits antimicrobial activity. These properties are beneficial for a wide range of already commercial applications such as drug delivery, wound dressings, and skin-tissue engineering, as a coagulant, flocculant or adsorbent in wastewater treatment, and as a food additive and dietary fibre [4,5,6]. Its ability to form films and its biocompatibility and biodegradability promotes the use of chitosan as a single-use packaging material [1,7], which is the focus of this study.

Also perceived to be suitable for such applications is alginate, which consists of linear co-polymers of (1-4)-linked β-D mannuronic acid and α-L-guluronic acid [8]. It can be isolated from brown algae such as *Laminaria digitata*, *Laminaria japonica*, *Laminaria hyperborea*, *Macrocystis pyrifera* and *Ascophyllum nodosum*. Alginate is used commercially in the food and pharmaceutical industry as a thickening, gel-forming and stabilization agent; however, its versatility has not yet been fully exploited. Recent studies suggest the use of alginate in biomedical applications (drug delivery, wound dressing, tissue regeneration), the food industry (encapsulation, functional food) and as film packaging [9,10].

Cellulose nanocrystals (CNCs), commonly extracted by acidic hydrolysis of cellulose, have been applied as a reinforcement agent to biopolymer-based films to improve mechanical and barrier properties, bringing such materials closer to achieving protection and user experience comparable to commercial plastic foils and wrappings [11,12,13]. The advantage of CNCs lies in the worldwide availability of the raw material, economical accessibility, high mechanical strength, high specific surface, biocompatibility, and biodegradability. Several studies have reported the increase of tensile strength upon the addition of CNCs into the chitosan matrix [14,15,16]; however, the uniform dispersion of CNCs in the matrix is crucial for the production of a large matrix/nanofiller interfacial area that is responsible for changing molecular mobility and consequently improving mechanical properties [17]. Although CNCs have shown good compatibility with alginate-based films [18,19], significantly improving mechanical strength, their numerous hydroxyl groups on the surface are the reason for their hydrophilic character that induces lower compatibility with non-polar or positively charged polymer matrices, such as chitosan. The authors in Dong et al. [14] and Rubentheren et al. [20] observed the improvement of mechanical strength in chitosan-based films upon CNC integration that increased gradually with the concentration of incorporated CNCs; however, the water barrier of such films was not evaluated in these studies. The homogeneous dispersion of CNCs in the chitosan matrix can be inflicted mechanically by homogenization [21] and microfluidization [22] or by the surface modification of CNCs. The latter is frequently implemented in polymer chemistry, e.g., the authors in Grunert and Winter [23] functionalised the surface of CNCs through silylation and incorporated them into cellulose acetate butyrate matrix; the authors in Lin et al. [24] acetylated CNCs, which improves their compatibility with poly(lactic) acid; and the authors in Vasconcelos et al. [25] applied carboxymethylation, which improved the solubility of CNCs in polar media. However, the use of modified CNCs is not as widespread in biopolymer applications. Regarding chitosan-based films, the incorporation of CNCs was ensured either mechanically or by surface modification such as functionalisation with methyl adipolychloride [26] and treatment with O_2_/laccase/TEMPO [13], which were shown to be successful.

The main advantage of such biocomposites is their renewable origin, non-toxicity, and biodegradability. However, currently, the commercial use of alginate, chitosan and CNC biocomposites is not yet viable due to a lack of technology for cost-effective industrial production as well as insufficient knowledge of their behaviour in real-life applications such as in environments with high or low humidity [27,28]. To push such biocomposites closer to commercial use as biodegradable packaging, understanding the impact of environmental conditions (such as environmental humidity) on the performance of the film is essential, as it affects moisture content and thus mechanical properties. The structure of chitosan that is abundant in hydroxyl and amino functional groups, as well as glycosidic linkages, giving chitosan-based films a high affinity to water [29,30]. The presence of moisture in the film induces a plasticizing effect by interrupting hydrogen bonding between the oxygen in the remaining acetyl group and hydrogen in the hydroxyl group in chitosan chains, causing an alteration in mechanical properties [31,32]. Although da Silva et al. [33] investigated the relationship between drying technique, moisture content and mechanical properties in alginate-based films and Giz et al. [34] observed that the extent of stress relaxation increases with the increase of humidity, so far, there has been no systematic study of the mechanical, barrier and thermal properties of both chitosan- and alginate-based films reinforced with pristine and acetylated CNCs. Furthermore, to the best of our knowledge, there has been no research depicting the relationship between environment humidity and the mechanical properties of such biocomposites.

**Figure 1:**
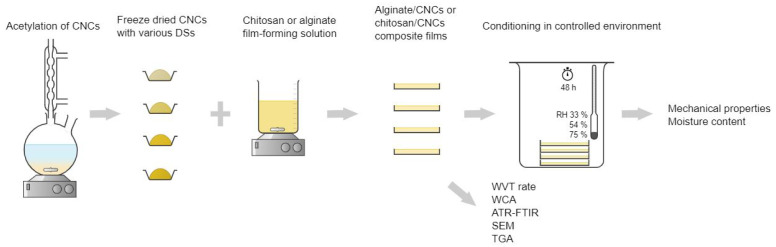
Schematic representation of the study.

With this in mind, chitosan- and alginate-based films with incorporated pristine and modified CNCs were investigated in terms of their suitability as single-use biodegradable packaging by evaluation of WVT rate, WCA, TGA, mechanical properties, ATR–FTIR and morphology. As acetylation is the most commonly used modification that is already carried out on an industrial scale for cellulose materials, this type of functionalisation was chosen for CNCs that were acetylated to three different degrees of substitution (determined by ATR–FTIR analysis) and then incorporated into chitosan and alginate film-forming solutions. The morphology of the fabricated films was observed with SEM, the chemical structure was inspected with ATR–FTIR, and the performance of the films for packaging purposes was assessed through the evaluation of moisture content (MC), tensile strength (TS) and elongation at break (ϵ) in dependency to environmental relative humidity (RH), as well as water contact angle (WCA), thermal stability and the ability to reduce water vapour transmission rate (WVT) as demonstrated in Figure 1.

## 2. Materials and Methods

### 2.1. Materials

Chitosan (high molecular weight, >85% deacetylated), lactic acid (85% aqueous solution) and sodium alginate were purchased from Sigma–Aldrich (Darmstadt, Germany). Glycerol, used as plasticizer, and cellulose nanocrystals (in 3 wt.% suspension) were bought from Pharmachem d.o.o (Ljubljana, Slovenia) and Navitas (Stari trg pri Ložu, Slovenia), respectively. CaCl_2_ (99.0–103.0%) and NaCl (≥99.5%, analytical grade), used in water-barrier studies and conditioning of the films, were acquired from Merck (Darmstadt, Germany), while MgNO_3_ × 6H_2_O (≥98.0%) was purchased from Honeywell (Charlotte, NC, USA). For the acetylation of CNCs, pyridine (Merck, Darmstadt, Germany), acetic anhydride (Sigma–Aldrich, Darmstadt, Germany) and acetone (Honeywell, Charlotte, North Carolina, USA) were used. Deionized water was used throughout the experiments unless stated otherwise.

### 2.2. Methods

### 2.3. Cellulose Nanocrystal Acetylation

A total of 1 g of lyophilized and crushed CNC powder was weighed into a round-bottom reactor. After the addition of 5 mL of pyridine, the mixture was sonicated for 15 min and heated to 90 °C. As the temperature stabilized, the reaction was started by the drop-wise addition of 5 mL of acetic anhydride. CNCs were subjected to the reaction for 60 min, 180 min and 300 min, and then washed in three consecutive cycles with water and with 50% acetone in water solution to remove any unreacted acetic anhydride. CNCs were then low-pressure freeze-dried prior to analysis and incorporation into the chitosan-based films.

### 2.4. Fabrication of Biopolymer/Acetylated CNCs Biocomposite Films

The films were fabricated according to the protocol described by Lavrič et al. [15]. Briefly, chitosan in the amount of 1.5 wt.% was dissolved in 1 *v*/*v*% lactic acid (prepared from ultrapure water, 18.2 MΩ cm) and stirred overnight on a magnetic stirrer (300 rpm) at room temperature together with 30% glycerol (based on biopolymer mass) to form a film-forming solution (FFS). The solution was vacuum-filtered through medical gauze to remove impurities. For chitosan/cellulose biocomposites, pristine CNCs and CNCs with different acetyl content were added in an amount to reach 3 wt.% with respect to chitosan. The mixture was homogenized at 6000 rpm for 3 min with an UltraTurrax homogenizer (IKA, Straufen, Germany). FFS was left on the laboratory counter overnight covered with para-film to eliminate air bubbles in the form of foam on the surface, which was collected with a spatula. A total of 50 g of FFS was carefully poured into a 12 × 12 cm petri dish. For alginate-based films, sodium alginate in an amount to reach 1.5 wt.% and glycerol (30 wt.% with respect to biomass) were dissolved in ultrapure water (18.2 MΩ cm) on the magnetic stirrer. Both pristine and modified CNCs were added in an amount to reach 5 wt.% based on the mass of alginate, as previously described in Lavrič et al. [15] and Huq et al. [35]. Again, 50 g of FFS was poured into a 12 × 12 cm petri dish. All samples were dried in the ventilated oven (Kambic, Slovenija) at 40 °C for 48 h and conditioned for 48 h in an airtight glass box at room temperature with RH controlled by CaCl${}_{2}$ (for RH 33%), saturated solution of MgNO${}_{3}$ × 6H_2_O (for RH 53%) and a saturated solution of NaCl (for RH 75%).

### 2.5. Physico-Chemical Properties

To determine the acetyl content in CNCs, ATR–FTIR analysis using Spectrum Two 135 (Perkin Elmer, Rodgau, Germany) was carried out in a wavenumber range from 400 cm^−1^ to 4000 cm${}^{-1}$ with a step of 4 cm${}^{-1}$ (accumulation of 32 scans for each step). The spectra were adjusted to the same baseline and normalized. The acetylation degree was calculated according to a ratio of I${}_{C=O}/$I${}_{C-O}$. The intensity I${}_{C=O}$ was correlated with the stretching of the acetyl group at 1740 cm^−1^, normalized to I${}_{C-O}$ at 1060 cm${}^{-1}$ which is unaffected by the reaction [36].

To observe pristine and modified CNCs under SEM, lyophilized CNCs powders were dispersed in water (approximately 0.05 wt.%) and sonicated for 10 min. A drop of the dispersion was placed on an aluminium holder that was previously polished with abrasive paper to smooth the surface and was left to dry. The samples were sputtered with a 2 nm layer of gold and inspected with a SUPRA 35V scanning electron microscope (Carl Zeiss, Jena, Germany) under near-vacuum conditions and with an accelerating voltage of 1 kV. For insight into the morphology of the fabricated films, they were cut to approximately 1 × 1 cm pieces, placed onto carbon tape and inspected under the same conditions as CNCs. Images were captured under magnification 10,000× and 20,000×.

The structure of the films was evaluated through ATR–FTIR with Spectrum Two 135 (Perkin Elmer, Germany) with wavenumber ranging from 400 to 4000 cm${}^{-1}$ with a step of 4 cm${}^{-1}$ (32 scans for each step).

Moisture content was determined using a Moisture Analyzer HE53 (Mettler Toledo, Columbus, OH, USA). Approx. 5 g of the sample was heated to 105 °C until constant mass. The difference between the initial and the final mass was accounted for by the moisture of the sample.

Film thickness was measured using an ABS Digital Thickness Gauge (Mitutoyo, Aurora, IL, USA).

For the determination of mechanical properties, samples were cut into pieces of length 6 cm and width 2 cm. Tensile strength (TS) and elongation at break ϵ were determined with an XLW Auto Tensile Tester (Labthink^®^ Instruments, Jinan, China) equipped with a 100 N loading cell. The gauge length segment was set to 4 cm and crosshead speed to 25 mm min${}^{-1}$. TS represents the ratio between maximal load and average initial cross-sectional area in the sample gauge segment, while ϵ is calculated by dividing the increase in length after the break-point with the initial gauge length.

For the evaluation of WVT, a film was fixed over a 15 mL glass flask filled with CaCl${}_{2}$ (previously activated at 105 °C for 2 h) with diameter 5 cm, with a top that had a carved circular area of 2.1 cm. These flasks were then subjected to the environment with 70% RH provided by saturated NaCl. For five consecutive days, the flask containing CaCl${}_{2}$ was weighed and *WVT*,expressed in g m${}^{2}$ 24 h${}^{-1}$ was calculated by Equation (1).

(1) $$WVT=\frac{\Delta m}{St}$$

where Δ*m* stands for an increase in mass (g), *S* represents the area of the film exposed to water vapour (1.84 × 10${}^{-4}$m${}^{2}$) and *t* is time in hours.

The evaluation of hydrophilicity/hydrophobicity was carried out through static WCA analysis, employing sessile drop measurement with a Tensiometer Theta T200 (Biolin Scientific, Darmstadt, Germany). The samples were cut into smaller pieces (approx. 2 × 3 cm${}^{-1}$) and fixed on a microscope slide. Ultrapure water (18.2 MΩ) with drop volume of 4 μL was used. The measurements were repeated at least five times per sample.

### 2.6. Thermal Stability

Thermogravimetric analysis was carried out in EGA 4000 (Perkin Elmer, Germany). Approximately 15 mg of the sample was subjected to thermal degradation under nitrogen atmosphere (flow rate was 30 mL min^−1^) in temperatures ranging from 50 °C to 700 °C with a rate of 10 °C min${}^{-1}$. The measurements were performed in duplicate.

### 2.7. Statistical Analysis

All results are reported as the mean value. A two-way analysis of variance (ANOVA) followed by Tukey’s test was applied using Origin 2018 (version b9.5.0.193) software to the data relating to mechanical properties to evaluate the impact of CNC acetylation and environmental humidity.

## 3. Results and Discussion

### 3.1. Acetylated Cellulose Nanomaterials

**Figure 2:**
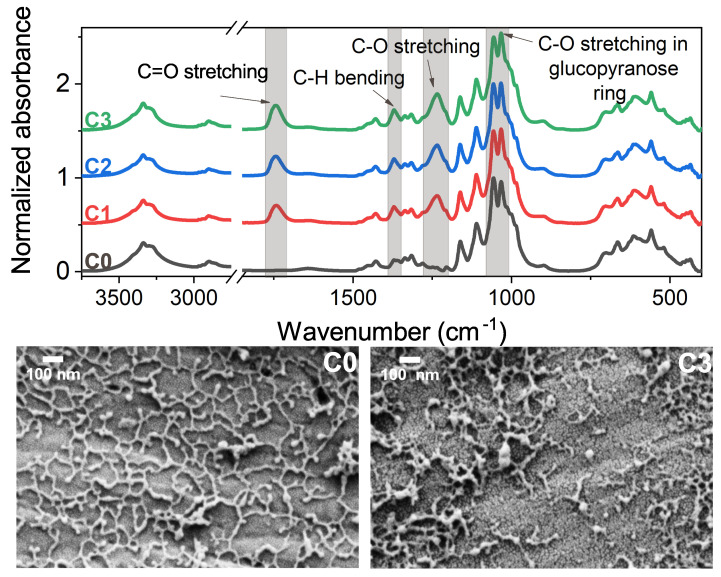
FTIR–ATR spectra of acetylated CNCs with C0 denoting the pristine material, C1, C2 and C3 representing samples with reaction time 1 h, 3 h and 5 h, respectively (upper part) and SEM micrographs of pristine (C0) and modified (C3) CNCs (lower part).

The ATR–FTIR analysis (Figure 2) of non-modified CNCs (labelled as C0) exhibits a characteristic peak for cellulose at 1070 cm${}^{-1}$ that is related to C-O stretching in the glucopyranose ring. The successful esterification of samples C1, C2 and C3 (denoting different reaction times and DS as presented in Table 1) was proven through the appearance of peaks centred at 1750 cm${}^{-1}$, 1370 cm${}^{-1}$ and 1250 cm${}^{-1}$ that are associated with C=O stretching, C-H bending and C-O stretching, respectively. The intensity of all of the newly formed peaks increases with reaction time. The FTIR spectra served as a basis for the calculation of DS indicated in Table 1. Pristine CNCs and samples modified to the highest DS were analysed under SEM (the lower part of Figure 2). In both samples, the particles are approximately 100 nm long and 14 nm wide; however, pristine CNCs tend to connect on their edges, forming a spider’s-web-like shape, which is not as pronounced in C3. This could be a consequence of surface modification that affects interparticle interactions.

### 3.2. Morphology of the Fabricated Polysaccharide-Based Films

**Figure 3:**
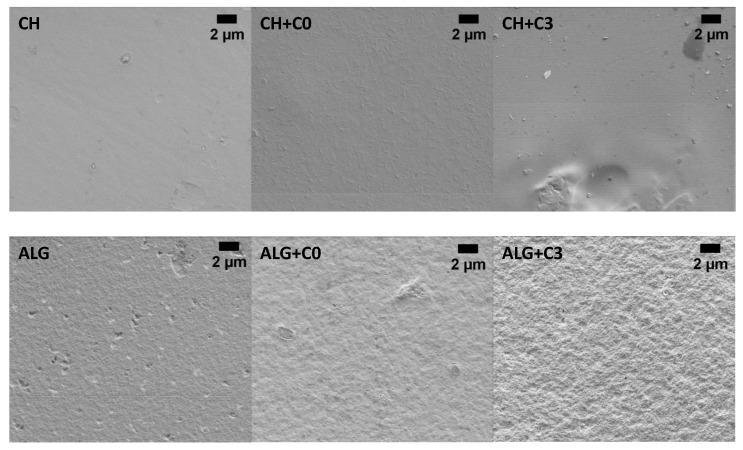
SEM micrographs of chitosan-based film (CH), chitosan films with pristine CNCs (CH+C0), chitosan films with acetylated CNCs (CH+C3) in the upper part and alginate-based film (ALG), alginate films with pristine CNCs (ALG+C0), alginate films with acetylated CNCs (ALG+C3).

The fabricated films were first inspected under SEM to obtain an insight into the morphology (Figure 3 and Figure S1). For clarity, the following labels apply for the films throughout the manuscript: CH, CH+C0, CH+C1, CH+C2 and CH+C3 for chitosan film; chitosan film with incorporated pristine CNCs; chitosan film with incorporated C1, C2 and C3, respectively; and ALG, ALG+C0, ALG+C1, ALG+C2 and ALG+C3 alginate-based films; the film consisting only of alginate was labelled ALG; alginate film with pristine CNCs was labelled ALG+C0; and films with incorporated C1, C2 and C3 were labelled ALG+C1, ALG+C2 and ALG+C3, respectively. The addition of CNCs into initially smooth CH film resulted in aggregates with an average length of 700 ± 200 nm and width of 80 ± 30 nm. It has to be noted that the size of an individual CNC is up to 6 nm in width and no more than 20 nm in length [37]. After incorporation of C3, no aggregates were visible on the surface of the film, pointing to the homogeneous incorporation of acetylated CNCs into the chitosan matrix. On the other hand, the surface of ALG appears rougher and more granulated, which does not change upon the addition of C0. However, the surface of ALG+C3 increases even further in roughness, indicating that the modified CNCs do not mix as well into the alginate matrix as pristine CNCs.

### 3.3. FTIR–ATR Analysis of the Fabricated Polysaccharide-Based Films

**Figure 4:**
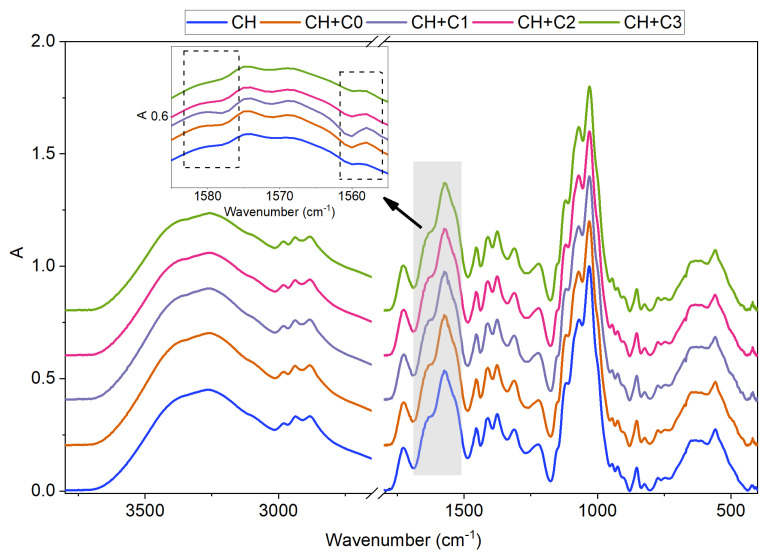
FTIR–ATR analysis of chitosan-based films with and without integrated CNCs of various DS.

Chitosan is a linear co-polymer consisting of D-glucosamine and N-acetyl-D-glucosamine, which is mirrored in its FTIR spectrum (the blue line in Figure 4) and exhibits a broad peak between 3500 cm${}^{-1}$ and 3000 cm${}^{-1}$ corresponding to N-H and O-H stretching and the peak between 3000 cm${}^{-1}$ and 2800 cm${}^{-1}$ associated with C-H bond stretching. The peaks appearing between 1750 cm${}^{-1}$ and 1500 cm${}^{-1}$ are correlated with -C=O- stretching (amide I, 1723 cm${}^{-1}$), -NH- bending (amide II, 1570 cm${}^{-1}$)) and -CN- stretching vibrations (amide III, 1377 cm${}^{-1}$) [32,38]. Upon the addition of CNCs (pristine as well as modified), only relatively minor changes in the spectra are observed. A peak located at 1068 cm ${}^{-1}$, corresponding to -C-O-C- stretching, increased due to -C-O-C- bridge vibrations of the glucopyranose ring. For better visibility, the regions between 1590 cm${}^{-1}$ and 1550 cm${}^{-1}$ in which slight changes occurred are magnified in Figure 4. The peak at 1580 cm${}^{-1}$, related to N-H bending in amines is slightly more pronounced in CH+C0 and CH+C1, and it decreases again with higher DS. Similarly, the peak centred at 1550 cm ${}^{-1}$ correlated with -N-O- stretching is increased in samples CH+C0, CH+C1 and starts to decrease again with CH+C2 and CH+C3. These observations could point to repulsive intra-molecular interactions between chitosan and CNCs that decrease with higher DS.

**Figure 5:**
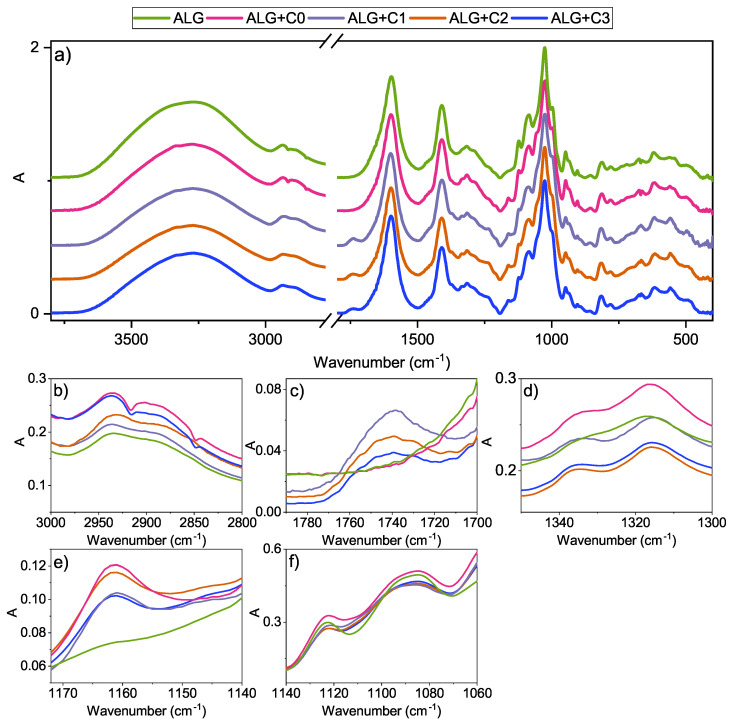
(**a**) FTIR–ATR analysis of alginate-based films with and without integrated CNCs of various DS, with subfigures (**b**–**f**) exhibiting regions where change upon addition of pristine or modified CNCs is observed.

Alginate, also a linear co-polymer, is made of β-D-mannuronate and α-L-guluronate, which are linked by β-1,4 and α-1,4 glycosidic bonds. In Figure 5a, full spectra of the samples are presented, and Figure 5b–f exhibit the fragments of the spectra where changes upon integration of pristine and modified CNCs occur. The blue line denotes the characteristic spectrum of ALG. A broad peak between 3700 cm${}^{-1}$ and 2980 cm${}^{-1}$, correlated to -O-H stretching, decreases in intensity, first slightly upon the incorporation of C0, and then further with the acetylation of CNCs. The peaks located between 2980 cm${}^{-1}$ and 2880 cm${}^{-1}$ are related to the asymmetric and symmetric stretching modes of -CH${}_{2}$- [39], and the wear peak centred at 2938 cm${}^{-1}$ is related to -CH- stretching (Figure 5b). Although the shape of the peak remained the same when incorporating pristine CNCs, the original bonds were disturbed by acetylated CNCs, whereas the peaks for -CH${}_{2}$- stretching shifted to lower wavenumbers, which points to a weakening of the bond [39], and the peak correlated with -CH- stretching disappeared. In the region between 1770 cm${}^{-1}$ and 1710 cm${}^{-1}$, Figure 5c, a peak corresponding to -C=O- stretching vibrations in acetylated CNCs emerges in samples ALG+C1, ALG+C2 and ALG+C3. Its intensity, however, is inversely proportional to the DS of CNCs. This could be attributed to the better integration of CNCs with lower DS in the alginate matrix, while a higher degree of acetylation leads to the aggregation of CNCs. Indicating the presence of cellulose, the peaks at 1335 cm${}^{-1}$ (related to -OH- bending) and 1160${}^{-1}$ (related to -C-O- stretching) can be observed in Figure 5d,e in samples with incorporated CNCs but not in ALG. Lastly, the peaks centred at 1123 cm${}^{-1}$ and 1086 cm${}^{-1}$ (Figure 5f) ascribed to -C-C- and -C-O- stretching vibrations, respectively, became less pronounced upon the reinforcement of alginate-based films with CNCs.

### 3.4. Physiochemical Properties of the Fabricated Polysaccharide-Based Films

**Figure 6:**
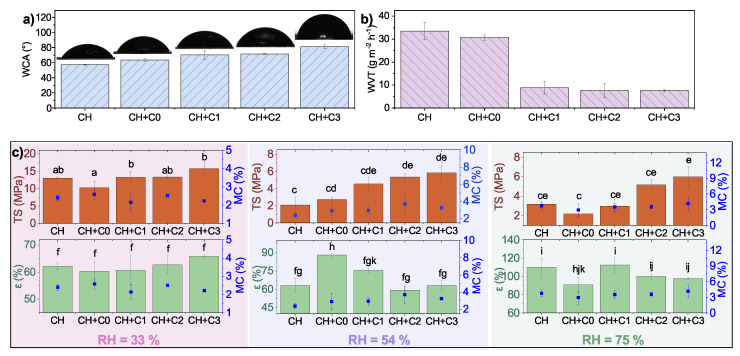
(**a**) Water contact angle (WCA), (**b**) water vapour transmission (WVT) and (**c**) mechanical properties with respect to environmental RH of chitosan-based films, where different letters denote samples with significantly different mean value (*p* < 0.05).

Water-related properties, such as WCA and WVT rate, are important properties in packaging materials for the protection of the product from external impact. In chitosan-based films, the addition of non-modified CNCs already caused an increase in WCA from the initial 57° to 64° (Figure 6a), which agrees with the literature [15]. This could be attributed to the change in morphology and formation of aggregates on the surface. Furthermore, acetylation decreases the surface free energy and thus hydrophilicity through the introduction of acetyl groups to the surface of CNCs, the WCA in samples CH+C1, CH+C2, and CH+C3 further increases (up to 81°). WVT rate decreases slightly upon the inclusion of pristine CNCs (from an initial 33 g m${}^{-2}$ h${}^{-1}$ ± 4 g m${}^{-2}$ h${}^{-1}$ to 30 g m${}^{-2}$ h${}^{-1}$ ± 1 g m${}^{-2}$ h${}^{-1}$). Sample CH+C3 exhibits a notable improvement in water vapour barrier—the WVT rate decreased as much as 77% from the initial value that was observed with the functionalisation of CNCs (Figure 6b).

The mechanical properties of biocomposite films are strongly dependent on environmental moisture, as they influence the MC. To simulate various environments, the films were conditioned in three different relative humidities (RH 33%, 50%, 75%). Regardless of the humidity, the samples with the modified CNCs exhibited the highest mechanical resistance compared to CH and CH+CNCs, which increased with DS (Figure 6c)—the highest TS was observed in sample CH+C3. The addition of pristine CNCs to the chitosan matrix resulted in a slight decrease of TS in comparison to CH films, which was attributed to the fact that pristine CNCs did not mix as homogeneously into the chitosan matrix as modified CNCS, and formed aggregates, as seen in Figure 3. At RH 54%, it slightly increased, but remained in the range of standard deviation. It was observed that the films exhibit higher TS in drier environments (RH 33%) and therefore lower MC in the film. However, the MC in the films seems to be independent of dopant as, in all samples conditioned at the same RH, the values are in the range of standard deviation (around 2.5% in RH 33%, 3.0% in RH 50% and 3.6% in RH 75%). Contrary to TS, elongation at break (ϵ) is not as closely related to the DS of modified CNCs and is higher in more humid environments. This is reasoned by the fact that moisture in the film (which increases along with RH) acts as a plasticizer and induces the stretchiness of the film by promoting the movement of biopolymer chains [32].

**Figure 7:**
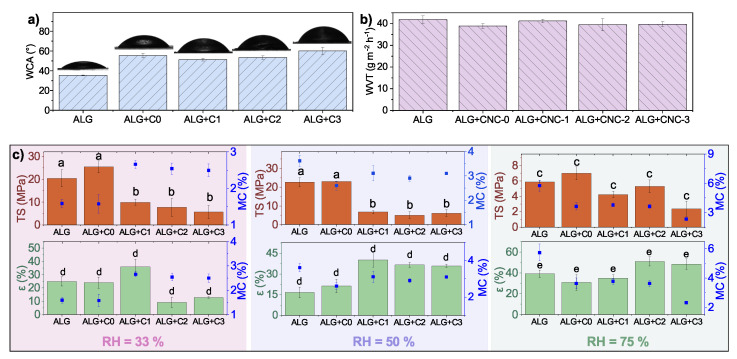
(**a**) Water contact angle (WCA), (**b**) water vapour transmission (WVT) and (**c**) mechanical properties with respect to environmental RH of alginate-based films, where different letters denote samples with significantly different mean value (*p* < 0.05).

Both alginate-based films and CNCs are very hydrophilic in nature. The initial alginate film (ALG) exhibits WCA of 35 ± 0.4°, and the addition of pristine and modified CNCs into the matrix slightly decreases hydrophilicity (up to 60 ± 3.6° in sample ALG+C3). Only minor differences in WCA are observed between samples ALG+C0, ALG+C1, ALG+C2 and ALG+C3, indicating that the change in water repellency is due to morphology rather than a change in free surface energy. The incorporation of CNCs into the alginate matrix does not alter the barrier properties. WVT through alginate-based films was measured to be about 40 g m${}^{-2}$ h${}^{-1}$ (from 39 g m${}^{-2}$ h${}^{-1}$ to 42 g m${}^{-2}$ h${}^{-1}$ with the difference in range of standard deviation) as shown in Figure 7b.

Contrary to chitosan-based films, mixing pristine CNCs into the alginate matrix slightly improves the TS of films, while it is significantly decreased by the incorporation of surface acetylated nanoparticles. This could be attributed to the fact that modified CNCs do not mix homogeneously into the alginate FFS and therefore form aggregates and cause the uneven surface of the film (seen in Figure 3). The trend of decreasing mechanical strength was observed in all three tested RHs. Again, the TS of the films is greatly influenced by environmental humidity and drops with an increase in RH. The films perform best in drier environments as the maximum TS was measured with sample ALG+C0, reaching 25 ± 3 MP in RH 33%. It was observed that MC remains rather stable in ALG+C1, ALG+C2 and ALG+C3, which could be explained by the formation of aggregates that induce pores in the film that hold the moisture in the film. The largest fluctuation in samples containing modified CNCs is 1% (in sample ALG+C1), while the MC in ALG samples is highly affected by environmental humidity and ranges from 1.6 ± 0.1% to 5.8 ± 0.6%. The trend is reversed in the environment with the highest humidity, where the samples doped with pristine or modified CNCs exhibit lower MC. This phenomenon could be attributed to the hydrophilic nature of alginate, binding water from the environment. ϵ shows less distinct trends among neat and reinforced alginate films. In RH 33% and RH 50%, sample ALG+C1 exhibits the highest elongation (36 ± 6% and 40 ± 5%, respectively), while in humid environments (RH 75%), the highest ϵ is observed in film doped with C1 (34 ± 3%).

### 3.5. Thermal Stability

**Figure 8:**
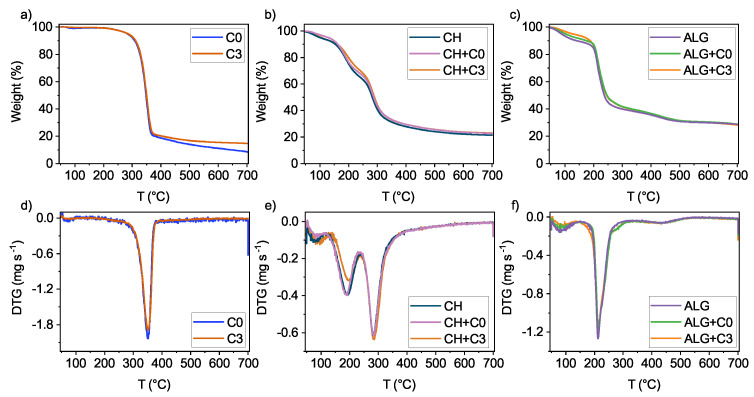
Thermal stability of (**a**) pristine and modified CNCs, (**b**) chitosan-based films (samples CH, CH+C0, CH+C3), and (**c**) alginate-based films (samples ALG, ALG+C0, ALG+C3) and derivates of their weight loss (**d**–**f**), respectively.

The thermal stability of biocomposite films was determined through the observation of the change in sample weight. The degradation of CNCs (both pristine and modified) occurs in a single stage that begins at 260 °C, as shown in Figure 8a,c and results in a loss of 80% of the initial mass. Chitosan-based films show degradation in three stages (Figure 8b,c), with the first one featuring a loss of free bound water, occurring between 60 °C and 140 °C. The second stage of degradation starts at around 140 °C and represents a loss of bound water in the films as well as degradation of shorter chains [40]. Here, a slight shift in the CH+C3 curve is observed, as it reaches maximum degradation 10 °C later than CH and CH+C0, pointing to the fact that the water is well bound into the matrix and a higher temperature is needed for it to evaporate. The last stage takes place after 250 °C and is related to the decomposition of chitosan polymer chains, with the residues accounting for 23% of initial mass in CH+C0 and CH+C3 and 21% in CH. The degradation of alginate-based films occurs in two stages. As with chitosan-based films, the loss of mass up to 120 °C accounts for the loss of free water (Figure 8c,f). The second stage starts at 160 °C and is responsible for the loss of bound water, as well as the decomposition of polymer chains. In samples ALG+C0 and ALG+C3, degradation to the final 28% of initial mass is slightly delayed compared to ALG, indicating the better thermal stability of films doped with CNCs.

## 4. Conclusions

Pristine and acetylated CNCs with various DSs (0.299, 0.334 and 0.399) were incorporated into chitosan and alginate matrices to fabricate biocomposite films. Properties relevant to single-use packaging applications, such as WVT rate, wettability, thermal stability, moisture content and mechanical properties, were thoroughly investigated. Furthermore, the effect of environmental humidity on the latter was evaluated to obtain insight into the applicability of such materials in the real world. SEM investigation indicated that the incorporation of pristine CNCs induced the formation of aggregates in chitosan-based films due to their hydrophilic nature, while their acetylated counterparts mixed homogeneously into the film. On the other hand, in alginate-based films, the modification of CNCs produced a more structured surface that was not observed upon the incorporation of pristine CNCs. ATR–FTIR analysis of fabricated biocomposites revealed a slight change in the peak corresponding to -C-O-C- vibrations in chitosan-based films, while spectra of alginate-based films were altered in five different regions. In chitosan-based films, the modification of CNCs prior to their integration positively influenced WCA, WVT rate and mechanical properties, which was especially noticeable in environments with high humidity (RH 75%). On the contrary, in the alginate matrix, the best performance was observed in films with incorporated pristine CNCs, as they mix homogeneously into the matrix without the need for modification. Overall, films exhibited the highest mechanical strength in dryer environments, whereas elongation was higher in more humid environments, which is in line with the published literature [41,42]. The results of this study show that a higher degree of acetylation of CNCs (DS = 0.399) prior to incorporation into the chitosan matrix improves properties relevant for packaging applications, and this pretreatment is not needed in alginate-based biocomposites. Such materials could be suitable for packing dry products.

## Figures and Tables

**Table 1:** Labelling of the CNC samples according to reaction time and their corresponding calculated DS.

Sample	Reaction Time [min]	DS
C0	0	0
C1	60	0.299
C2	180	0.334
C3	300	0.399

## Data Availability

The data that support the findings of this study and materials are available from the corresponding author upon reasonable request.

## Supplementary Materials

The following supporting information can be downloaded at: https://www.mdpi.com/article/10.3390/polym15071663/s1, Figure S1. SEM micrographs of alginate films with pristine CNCs (ALG+C0), alginate films with acetylated CNCs (ALG+C3), chitosan films with pristine CNCs (CH+C0), chitosan films with acetylated CNCs (CH+C3) in the upper part.
