# Supplementary material for: Effect of Environment on Acetylated Cellulose Nanocrystal-Reinforced Biopolymers Films
Source: Polymers (Basel). 2023 Mar 27;15(7):1663. doi: 10.3390/polym15071663 (PMC10096506; doi:10.3390/polym15071663)
Supplement: Supplementary file 1 [file polymers-15-01663-s001.zip › polymers-2275412 supplementary.pdf]

Supplementary

Effect of Environment on Acetylated Cellulose Nanocrystal-Reinforced Biopolymers Films

Ana Oberlintner [1,2], Blaž Likozar [2] and Uroš Novak [1,*]

[1] Department of Catalysis and Chemical Reaction Engineering, National Institute of Chemistry, SI-1000 Ljubljana, Slovenia

[2] International Postgraduate School Jožef Stefan, SI-1000 Ljubljana, Slovenia

* Correspondence: uros.novak@ki.si

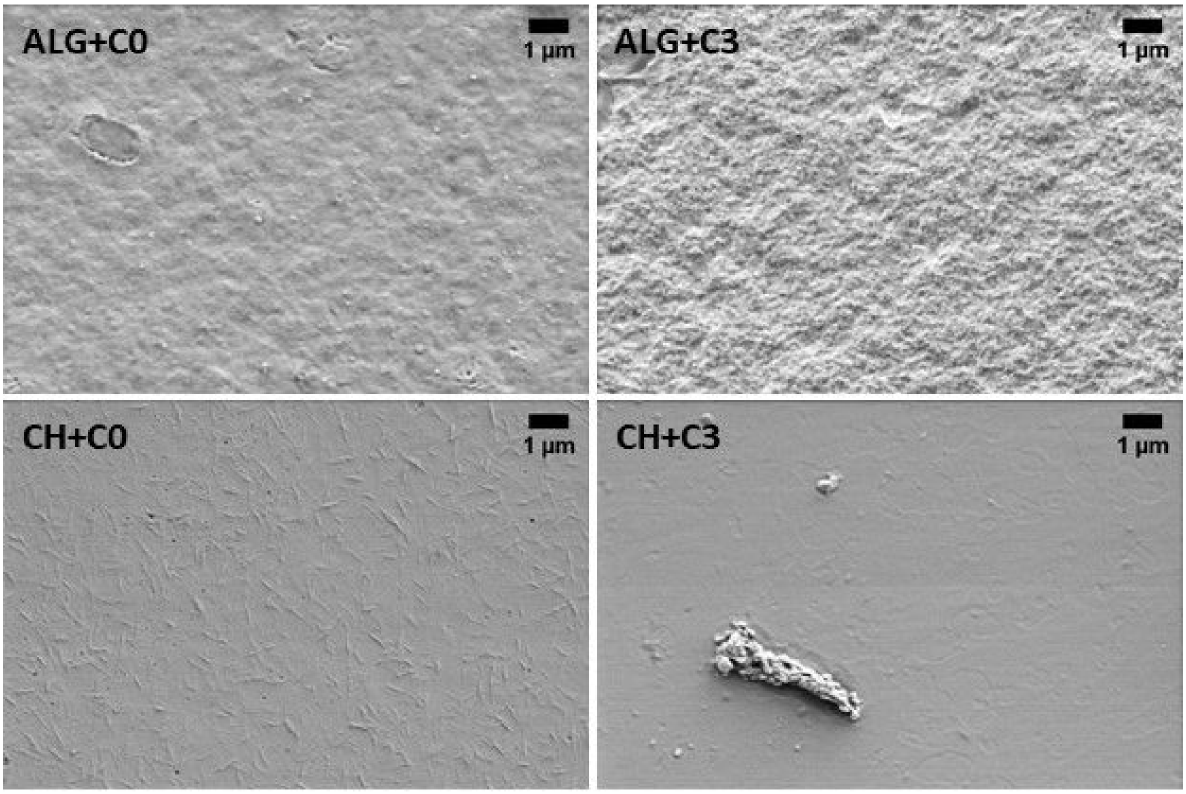

Figure S1. SEM micrographs of alginate films with pristine CNCs (ALG+C0), alginate films with acetylated CNCs (ALG+C3), chitosan films with pristine CNCs (CH+C0), chitosan films with acetylated CNCs (CH+C3) in the upper part.
